# Direct application of non-thermal plasma technology for the elimination of biofilm from endoscope channels

**DOI:** 10.1038/s41598-025-21136-w

**Published:** 2025-10-23

**Authors:** Elisabeth A. Slade, Gillian E. Clayton, George Hodgkins, Darren M. Reynolds, Chris Hancock, Robin M. S. Thorn

**Affiliations:** 1 School of Applied Sciences, College of Health, Science and Society, University of the West of England, Bristol, UK; 2 Institute of Biomedical and Environmental Health Research (IBEHR), School of Computing, Engineering and Physical Sciences (CEPS), University of the West of Scotland, Paisley, UK; 3 Creo Medical Ltd, 2 Riverside Court, Riverside Rd, Bath, UK; 4 School of Computer Science and Electronic Engineering, Bangor University, Bangor, UK

**Keywords:** Diseases, Medical research, Microbiology

## Abstract

Endoscopy procedures result in contamination of endoscope devices with potentially infectious organisms derived from the patient. Flexible endoscopes are complex, containing channels and ports that are difficult to clean and disinfect, further complicated by the presence of heat sensitive components, preventing use of high temperature sterilisation. Failure to effectively remove contamination from the internal channels and ports has the potential to result in microbial transmission between patients and subsequent outbreaks of infection. Non-thermal plasma (NTP) is generated by supplying a neutral gas with a continuous source of energy, resulting in the generation of reactive species, charged particles, electrons and ultraviolet (UV) photons, that have been shown to exhibit an antimicrobial effect. This study investigated the efficacy of a novel low energy NTP treatment system coupled with a prototype endoscope plasma applicator for the removal of viable biofilm contamination from the internal lumen of surrogate endoscope channels in an in vitro endoscope biofilm model. The results demonstrated that NTP can completely eliminate biofilms of *Pseudomonas aeruginosa*, *Staphylococcus aureus*, *Escherichia coli* and *Klebsiella pneumoniae* from surrogate endoscope channels. Therefore, NTP treatment could provide an alternative decontamination technology for reprocessing endoscope devices, to eradicate biofilm and eliminate the risk of cross infection between patients.

## Introduction

 More than two million endoscopy procedures are performed each year across 508 endoscopy units in the UK^1^. Demand for endoscopy services is increasing, both for assessment and treatment of symptomatic patients and for routine cancer screening^1^. The nature of endoscopy procedures results in contamination of the endoscope device with high levels of potentially infectious organisms derived from the patients native flora^2^. Flexible endoscopes contain several channels and ports that are difficult to clean and disinfect, and they usually cannot be sterilised at high temperatures^3^. Decontamination of endoscopes is a multi-step process that requires thorough manual cleaning, including brushing and flushing of all the accessible channels. Subsequently, devices undergo an automated cleaning and disinfection process in an endoscope washer disinfector (EWD) and drying using HEPA filtered air^4^. In the UK, decontamination of endoscopes must be undertaken within a dedicated endoscope reprocessing unit adhering to stringent guidelines dictated by the government Department of Health and Social Care. These guidelines detail requirements for design and installation of the reprocessing unit, procurement of EWDs, validation and verification of reprocessing parameters and the test methods employed^5–8^. Nevertheless, outbreaks of endoscope associated infections are widely reported^9–12^. Reprocessing failures resulting in microbial transmission between patients has been attributed to the ability of bacteria to form biofilms inside endoscope channels, especially where the internal channel surface becomes worn or damaged^3,13^, or where there has been insufficient drying leading to pooled liquid that can consequently promote microbial growth^14^. For example, following an endoscope associated outbreak of *P. aeruginosa* sepsis, inspection of endoscope channels revealed the presence of structures consistent with biofilm formation^3^. It has been highlighted that endoscope reprocessing failures and the potential for transmission between patients is likely to be far more common than the frequency of reported outbreaks suggests^15^. The study demonstrated that 47% of endoscope channels tested were culture positive, whereby isolated organisms included both environmental organisms and potential pathogens such as *Pseudomonas aeruginosa*, *Klebsiella pneumonia*, and *Escherichia coli*. In addition, biofilm was identified on all endoscope channels imaged using scanning electron microscopy and confocal scanning laser microscopy^15^.

A range of different sterilisation approaches have been developed to try and address the known issues with endoscope reprocessing. Ethylene oxide (EO) gas sterilisation of endoscopes has been used to control outbreaks, and it has been suggested that EO sterilisation should be widely adopted^12^. However, EO sterilisation is costly, and time consuming, so may require endoscopy units to purchase additional scopes to account for extended reprocessing time. In addition, there have been reports that EO sterilisation may result in damage to endoscopes over time and there are concerns over safety risks to reprocessing facility staff due to toxicity^12^. Automated liquid chemical sterilisation (LCS) approaches, commonly using peracetic acid, have been developed to address some of these issues, particularly enabling rapid cycle reprocessing times (typically ~ 30 min)^16^. However, there is a key requirement for initial effective removal of organic bioburden that can reduce treatment efficacy and complex geometry devices such as endoscopes can prove challenging to sterilise due to the requirements for liquid access to all regions of the scope (relying on probabilistic models)^17^. Vaporized hydrogen peroxide (VHP) sterilization is also used, demonstrating significant broad spectrum antimicrobial activity against a range of potential pathogens^18^, without the requirement for liquid delivery while maintaining shorter treatment cycle times. Nevertheless, limitations with this approach still exist, in that residual moisture within endoscopes can cause cycle failure due to the water freezing under the required vacuum, and/or leading to condensation of VHP into liquid hydrogen peroxide leading to reduced efficacy during treatment cycles^19^. Therefore, an alternative, low temperature decontamination technology to eradicate biofilm contamination that is safe, cost effective and minimises extended reprocessing time is required to eliminate reprocessing failure and consequently the risk of cross infection between patients.

Non-thermal plasma (NTP) is a partially ionised ‘fourth-state’ of matter, that is achieved by supplying a neutral gas (e.g. argon) with a continuous source of energy^20^. NTP exist in strong non-equilibrium, with a highly elevated electron temperature, while the temperature of neutral particles and overall gas temperature remains low. This results in a plasma ‘plume’ that is tolerable to living organisms, unlike thermal plasmas that are hundreds or thousands of degrees Celsius^21^. Generation of non-thermal plasma results in the generation of a number of reactive compounds, including charged particles, electrons and ultraviolet (UV) photons (usually far-UVC; 200–222 nm), that have been shown to exhibit antimicrobial properties^22,23^.

In recent years NTP technologies have been investigated for a wide range of applications. For example, NTP has been successfully trialled within the agri-food sector for the decontamination of fresh produce to remove potential pathogens and improve shelf-life^24,25^, and as an alternative sterilisation method for the dairy industry, to maintain quality and extend the shelf-life of milk through inactivation of pathogens and spoilage organisms^26^. Within wastewater processing NTP provides a means to not only inactivate microbial contamination, but the plasma generated reactive oxygen and nitrogen species also have the potential for degradation of organic and inorganic pollutants without the formation of secondary contaminants^27^. NTP has also been trialled in the medical field; for example for treatment of chronic wounds, resulting in a significant reduction in the bacterial load compared to untreated controls^28,29^; and for removal of biofilm from implanted medical devices (e.g. dental implants, joint replacements, pacemakers and heart valves)^30–32^. In addition, some automated NTP systems have been developed (e.g. V10 Air Plasma Sterilizer) that enable sterilisation of medical equipment, but this has only been applied within the veterinary sector and does not enable direct NTP application to target surfaces. A recent study demonstrates the capability of NTP treatment to significantly reduce bacterial biofilms cultured on the surface of titanium discs, further suggesting potential applications for treatment of biomaterial associated infections^30^. The mechanism of antimicrobial activity of NTP is thought to be multifaceted and to include, permeabilization of the cell membrane and cell wall leading to cytoplasm leakage, oxidative damage to cellular proteins and DNA caused by reactive oxygen and nitrogen species, and UV degradation of DNA^23^.

Given the versatility of non-thermal plasma for the decontamination of a variety of matrices, including solid surfaces colonised with bacterial biofilms, it is feasible that this technology can be adapted to successfully remove biofilm contamination from the internal surfaces of medical endoscope channels. Therefore, the main aim of this study was to evaluate the antimicrobial efficacy of a novel low energy non-thermal plasma treatment system coupled with a prototype endoscope plasma applicator (developed by Creo Medical Ltd., Chepstow, UK) for the removal of viable biofilm contamination from the internal lumen of an in vitro endoscope biofilm model (EBM). The novel NTP treatment system creates a non-thermal plasma through ionisation of argon gas, struck by radio frequencies (RF) and maintained with microwave energy, whereby the plasma jet is focused at the endoscope plasma applicator tip of a dielectric tube, enabling the plasma to be passed down endoscope operating channels.

## Materials and methods

### Preparation and maintenance of bacterial cultures

Bacterial cultures were maintained on beads at -80 °C, resuscitated as required on Nutrient Agar (NA) and incubated aerobically at 37 °C. Working cultures were stored on sealed plates at 4 °C. The following bacterial strains were used during this study; *Escherichia coli* NCTC 13919, *Klebsiella pneumonia* NCIMB 13281, *Pseudomonas aeruginosa* ATCC 15442, and *Staphylococcus aureus* NCTC 6538.

### Growth of bacterial biofilms in an endoscope biofilm model (EBM)

**Fig. 1:**
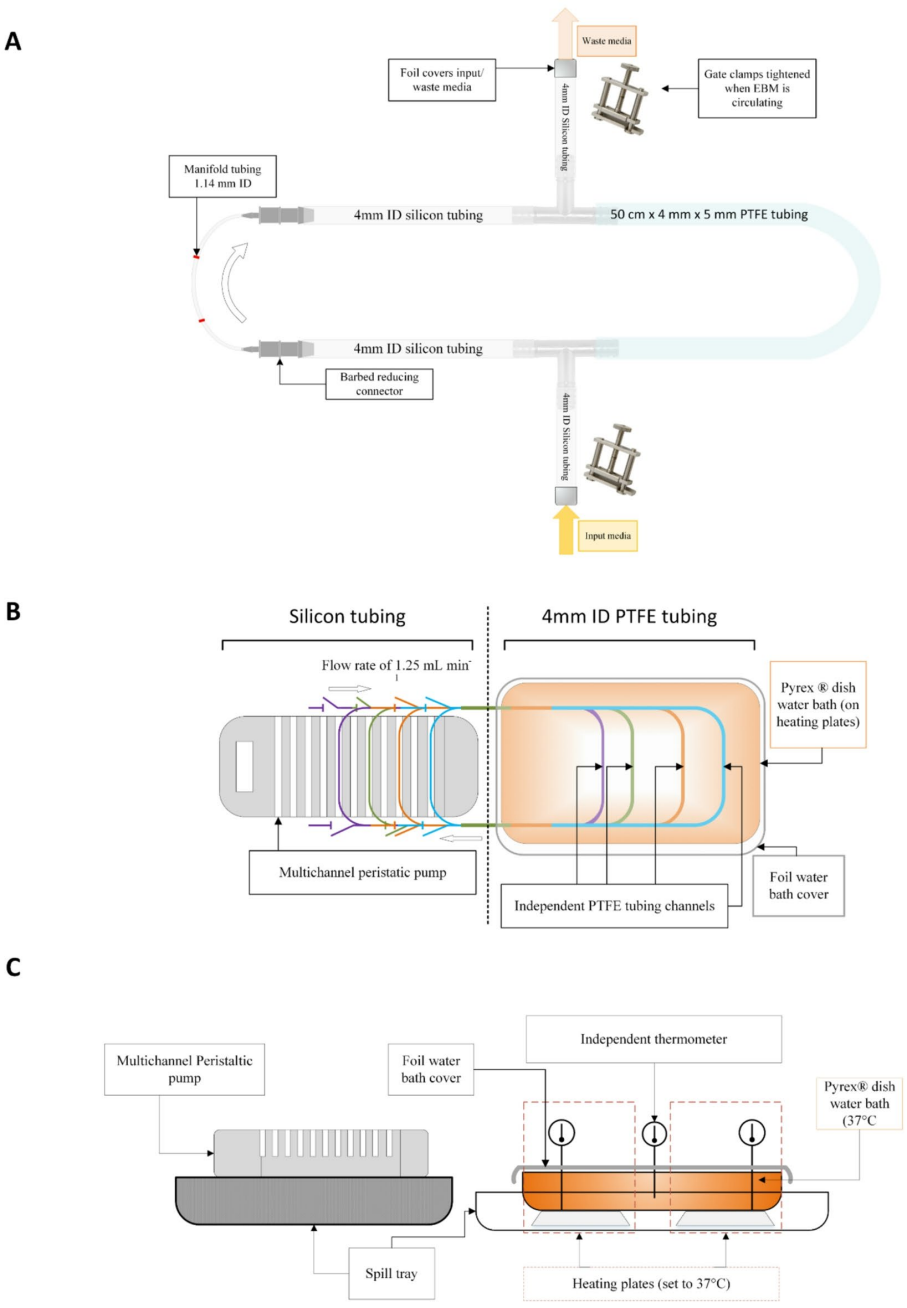
Endoscope Biofilm Model. [**A**] Schematic of the silicon/PTFE tubing loop with channels for the addition (input) and removal (waste) of media. Silicon tubing was stepped-up/-down with barbed reducing connectors. Polypropylene “T” connectors were used for isolating input/waste media tubing, and were clamped throughout circulation with gate clamps, with the open ends covered with foil. [**B**] Aerial schematic view of the endoscope biofilm model, [**C**] Profile schematic view of endoscope biofilm model. A multichannel peristatic pump, set to a flow rate of 1.25 mL min^- 1^, enabled continuous circulation of inoculated through 50 cm PTFE tubing sections (as surrogate endoscope operating channel) within closed tubing loops. PTFE tubing sections (within 3.7 L Pyrex® water bath) were kept at 37 °C. Foil was used to cover the water bath to reduce heat loss and evaporation.

Single species bacterial biofilms were cultured in an Endoscope Biofilm Model (EBM; see Fig. 1), based on a previously described method^33^. Polytetrafluoroethylene (PTFE) tubing 50 cm x 4 mm x 5 mm (length x internal diameter x outer diameter) was formed into a loop connected to peristaltic pump manifold tubing by two short sections of silicone tubing. T-pieces were used to connect two additional sections of silicone tubing, enabling input of inoculated or sterile broth and removal of waste culture media without disassembling the loop (see Fig. 1A). The entire tubing loop was sterilised by autoclaving prior to use.

Overnight plate cultures (18–24 h) were used to prepare 0.30 OD_600nm_ suspensions of the test organisms in 20 mL of nutrient broth (NB), resulting in a test suspension of ~ 3 × 10^8^ CFU mL^− 1^. The test suspension was transferred into the EBM tubing loop using a sterile 20 mL syringe. The entire tubing loop was filled, requiring approximately 10 mL of the test suspension. The ends of the input and waste tubing sections were covered with foil and the tubing isolated with gate clamps (Fig. 1A). The PTFE tubing sections were incubated by submergence in a waterbath at 37℃, with the manifold tubing connected into a multi-channel peristaltic pump to enable circulation of the inoculated media at a flow rate of 1.25 mL min^− 1^ (Fig. 1B and C). The EBM was incubated for a total of 72 h. At 24 and 48 h, circulation of media was paused, the nutrient broth was drained from the tubing loop using a sterile syringe (via the waste media tubing section), and the loop refilled with fresh sterile nutrient broth.

After 72 h, media circulation was stopped and the manifold tubing unclamped from the peristaltic pump. The liquid media was drained from the tubing loop using a sterile syringe. The full tubing loop was rinsed with 15 mL quarter strength Ringer’s solution to remove planktonic cells, using a pipette filler adjusted to the lowest dispensing setting. The PTFE tubing section was removed from the tubing loop and cut into sections for recovery and enumeration of the cultured biofilm and/or testing of non-thermal plasma treatments. Five cm at each end of the PTFE tubing was discarded, while the remaining 40 cm was retained and cut in to four test 10 cm PTFE tubing sections for subsequent experimentation.

### Non-thermal plasma treatment system

**Fig. 2:**
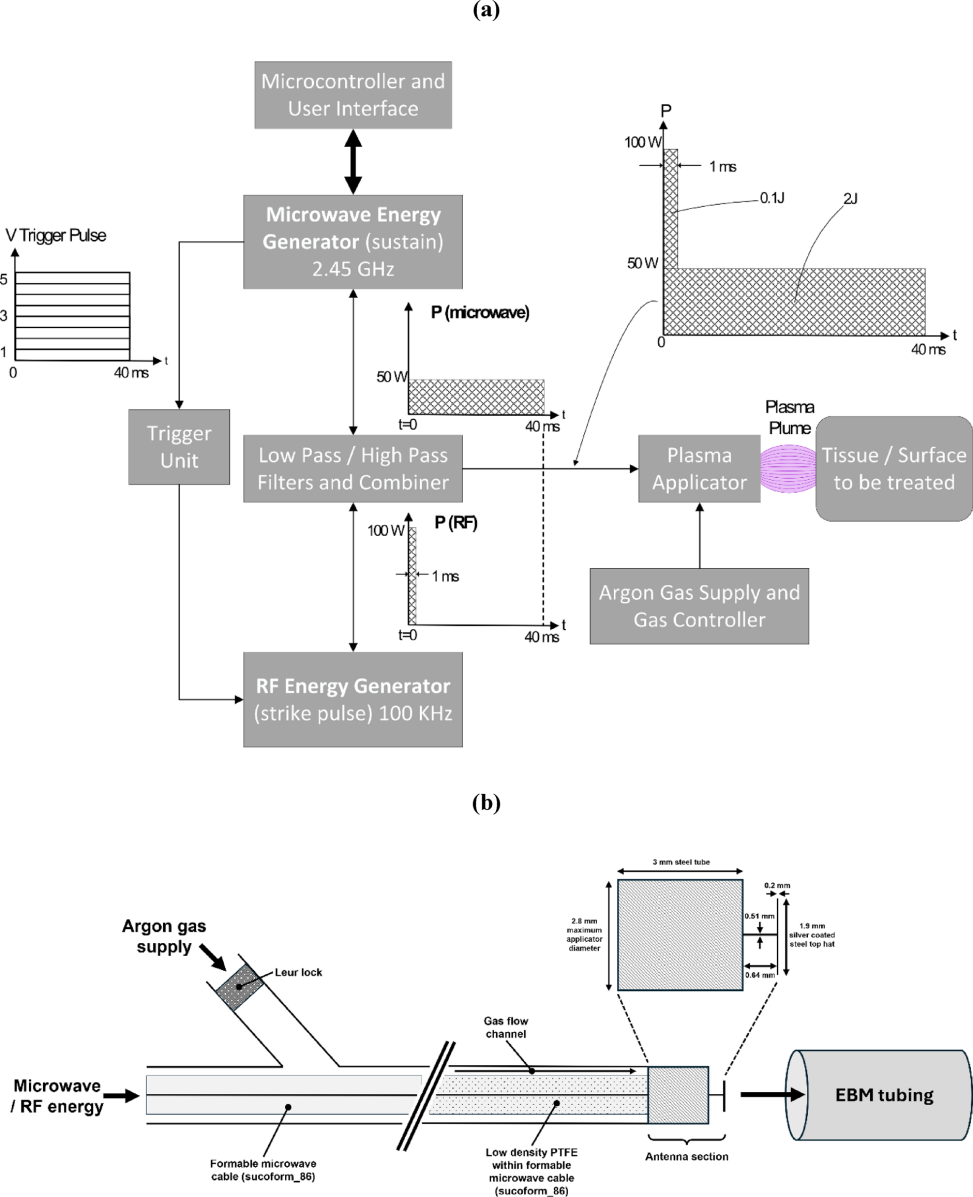
**(a)** Block Diagram showing the configuration of the non-thermal plasma treatment system. Indicating the layout of components including both the microwave energy and radio frequency (RF) energy sources, the microcontroller and user interface, power combiner with low pass and high pass filters, the applicator device and argon gas supply. **(b)** Schematic of the plasma applicator used to direct microwave or RF from the energy generator to the surface being treated. The applicator enables argon gas to be brought into close proximity with the generated plasma within a bespoke antenna section that encompasses a silver coated steel top hat antenna design at the distal end where the gas flows to produce a circumferential plasma that couples with the inner surface of the EMB tubing. The applicator can then be advanced into the EBM tubing via a motorised applicator driver at a defined speed.

Figure 2a shows a block diagram of the non-thermal plasma treatment system comprised of a microwave energy plasma sustain source and an RF energy plasma initiation strike unit. The microwave source operates at a spot frequency of 2.45 GHz that can produce up to 100 W of continuous wave (CW) power when driving into a load impedance of 50 Ω. The RF unit is set-up to produce a burst of high voltage sinusoidal waves with a peak amplitude of 1050 V when driving power to a load with impedance of 5.1 KΩ. The source is operating at a frequency of 100 kHz and the burst of RF is synchronised to the rising edge of the microwave pulse. The burst of RF energy is gated to 1ms, which is long enough to initiate the plasma. The microwave pulse (or burst of sinusoidal waves at a higher frequency of 2.45 GHz) is much longer than the burst of RF and is the dominant energy source that is impedance matched to the plasma (i.e. 40ms+). A trigger unit comprising a high-speed detector diode to detect the rising edge of the microwave signal, a comparator to ensure all levels of microwave signal down to a set threshold of 0.5 V, produces a 5 V amplitude. This voltage is then put through an analog differentiator, and a second comparator is set up to produce a pulse of duration 1ms when the output signal from the differentiator is compared with a reference voltage. The 1ms pulse is limited in amplitude to 4.7 V using a Zener diode and is fed into a logic AND gate that has the 1ms 4.7 V amplitude pulse on one input and a free running 100 kHz sinusoidal oscillator with a peak amplitude of 5 V on the second input to the AND gate. The synchronised 1ms pulse of 100 kHz RF from the trigger unit is fed into a suitable power MOSFET gate driver. The power MOSFET, with an appropriately sized voltage transformer comprised of a ferrite ring with 3 turns of enamelled copper wire on the primary side and 120 turns of enamelled copper wire on the secondary, is used to increase the drain voltage by a factor of 40 to ignite the plasma (shown in Fig. 2a). The initiation of the microwave sustain source comes from a microcontroller that forms a part of the user interface, to allow the user to input the desired amplitude of the microwave power, the ON and OFF times of the microwave pulse, and the total treatment time. The user interface also displays the impedance match between applicator and the plasma in to give an indication of the amount of microwave power being delivered into the plasma (a value of 10 dB indicates that 90% of the available microwave power is coupled into the plasma, thus delivered into the bacterial load). A power combining unit is connected between the two sources to allow both the burst of high voltage 100 kHz and the 2.45 GHz energy to be delivered down a single co-axial cable assembly to the applicator structure (shown in Fig. 2b). The power combining unit contains a low pass filter to let the 100 kHz energy through and stop the 2.45 GHz energy from damaging the 100 kHz source, and a high pass filter to let the 2.45 GHz energy through, but stop the 100 kHz energy from damaging the 2.45 GHz source.

The plasma is generated in a flexible co-axial applicator (Fig. 2b) made based on a Sucoform 86 cable assembly structure, which contains a centre conductor made from steel, copper and silver-plated wire, a low-density PTFE dielectric and a copper, tin plated tin-soaked braided outer. A PTFE tube with an outer diameter of 2.75 mm and wall thickness of 0.2 mm provides enough space for argon gas to flow between the outer diameter of the co-axial cable and the inner diameter of the PTFE tube. The distal end of the device contains a 3 mm long stainless-steel tube with an outer diameter of 2.8 mm that connects to the PTFE tube that delivers argon gas. The distal end of the Sucoform 86 co-axial cable terminates at the distal end of the 3 mm stainless steel tube, and the centre conductor of the co-axial cable extends from the distal end of the microwave cable a distance of 0.64 mm. A circular disk made from silver coated steel is connected to the end of the centre conductor. The dimensions of this disk are 1.9 mm diameter and 0.2 mm thick. This arrangement is known as a ‘Top hat’ antenna, where the 1.9 mm disk produces a capacitance and the 0.64 mm feed conductor forms an inductor. This produces a resonant circuit with a resonant frequency of 2.45 GHz. The 3 mm stainless steel tube also ensures that the argon gas can flow around the region of the ‘Top hat’ antenna to produce a circumferential plasma. The 1050 V peak 100 kHz sinusoidal voltage burst sets up a high enough electric field to strike the plasma, and the microwave source produces up to 100 W of 2.45 GHz energy to sustain. It should be noted that the microwave energy dominates the RF energy by a factor of at least 20 – this is illustrated in the system diagram given in Fig. 2a. The flow rate of the argon gas within the applicator is up to 10 L min^− 1^ at atmospheric pressure, although within this study this was limited to between 2 and 5 L min^− 1^.

### Non-thermal plasma treatment of endoscope biofilm model

Target microorganisms grown as biofilms within test 10 cm PTFE tubing sections (through culture in the EBM), were used to test the antibiofilm efficacy of the non-thermal plasma treatment system coupled with the prototype endoscope applicator. The non-thermal plasma treatment system was operated at various power settings from 80 W to 40 W, using a 28% duty cycle (i.e. plasma ON for 40 ms, OFF for 100 ms). Argon gas flow through the applicator was 2.5 L min^− 1^. The applicator was advanced through test tubing at a rate of 100 mm min^− 1^ for a total of 2 min (1 min forward, 1 min reverse), using a motorised applicator driver (developed by Creo Medical Ltd.). Non-thermal plasma treatment was compared to (1) ‘untreated’ control, (2) ‘applicator’ control; advancing the applicator without argon gas flow, (3) ‘argon’ control; advancing the applicator with (non-ionised) argon gas at a flow rate of 2.5 L min^− 1^.

### Recovery and enumeration of EBM biofilm

Prior to recovery, the external surface of PTFE tubing sections was decontaminated with 70% ethanol. After control or test treatments, 10 cm PTFE tubing sections were further sectioned to yield three replicate 1 cm pieces from each 10 cm section. Each 1 cm piece of PTFE tubing was then transferred to a 50 mL centrifuge tube containing 10 mL of sterile letheen broth (Remel Inc., San Diego, CA, USA). The biofilm was recovered from the internal surface of 1 cm PTFE tubing sections by vortex mixing each tube for 30 s, then sonicating for 1 min in a sonicating waterbath at 44 kHz, whereby this process was repeated three times. The resulting suspension was then serially diluted (10^− 1^ to 10^− 3^) in quarter strength Ringer’s solution and triplicate plated on to NA for enumeration. Agar plates were incubated at 37℃ for 18 to 24 h before counting. Prior to testing non-thermal plasma treatment, the biofilm formation throughout the length of PTFE tubing for each test species was characterised by recovery and enumeration from all four test 10 cm PTFE tubing sections to ensure even distribution of biofilm within the model system. Counts are reported as colony forming units (CFU) per cm^2^ (Log_10_ CFU cm^− 2^) of the internal surface of PTFE tubing.

### Complete biofilm elimination test

To determine if any biofilm cells, below the limit of detection for enumeration (< 0.9 Log_10_ CFU cm^− 2^), survived after the NTP treatment protocol, sterility testing was carried out. The outer surface of PTFE tubing sections was decontaminated with 70% ethanol, and the tubing was sectioned to yield three replicate 1 cm pieces from each 10 cm NTP treated section. Each 1 cm piece of PTFE tubing was transferred to a universal bottle containing 10 mL of sterile NB. Universal bottles were incubated at 37℃ and 150 rpm in a shaking incubator and examined for signs of bacterial growth (i.e. turbidity) for 7 days. Absence of growth was taken to demonstrate sterility of the test samples and confirmed by streak plating onto NA from each bottle after 7 days incubation.

### Data analysis

Data analysis was performed using GraphPad Prism 9. Two-way ANOVA with Tukey’s multiple comparisons test was used to calculate significant differences (1) between target species and (2) between tubing sections for a given species when quantifying biofilm growth within the EBM. For comparison of NTP treated biofilms with the various controls (see above) two-way ANOVA with Dunnett’s multiple comparisons test was used. A *p* < 0.05 was regarded as significant.

## Results

### Characterisation of endoscope biofilm model (EBM)

**Fig. 3:**
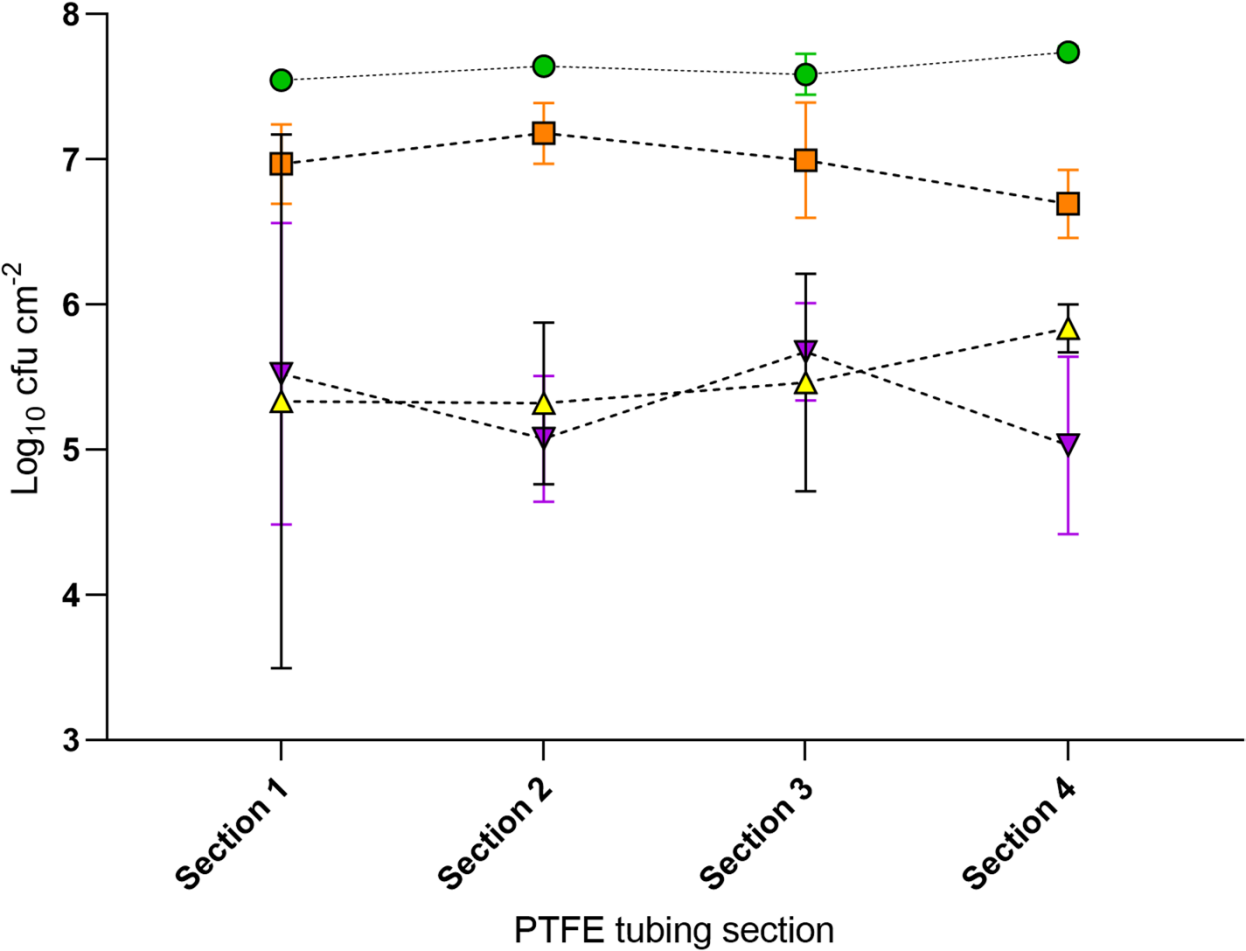
Characterisation of the endoscope biofilm model (EBM). Biofilm densities (log10 CFU cm^- 2^) of *Pseudomonas aeruginosa* (green filled circle), *Staphylococcus aureus* (orange filled square), *Klebsiella pneumoniae* (purple upside down filled triangle) and *Escherichia coli* (yellow filled traingle) after 72 h culture in the EBM with a flow rate of 1.25 mL min^- 1^ (*n* = 3 ± SD). Sections cut from 50 cm length of PTFE tubing used within the EBM. In the direction of flow, first 5 cm discarded, Sect. 1 = 5 cm to 15 cm, Sect. 2 = 15 cm to 25 cm, Sect. 3 = 25 cm to 35 cm, Sect. 4 = 35 to 45 cm, last 5 cm discarded. Within each section, three 1 cm subsections were processed for biofilm recovery.

The EBM method was a modified version of that developed by Vickery, Pajkos and Cossart (2004)^33^, whereby the media was replaced daily with fresh sterile broth in place of inoculated media, and the system was run for a total of 72 h. Therefore, there was a requirement to quantify and validate the biofilm density of all test species following 72 h of culture within the EBM (Fig. 3). Viable biofilm was recovered from the EBM for all four bacterial species tested; *P. aeruginosa*,* S. aureus*,* K. pneumonia* and *E. coli* (Fig. 3). Importantly, for *S. aureus*, *E. coli* or *K. pneumoniae* there were no significant differences in the biofilm density between the four test 10 cm PTFE tubing sections (Fig. 3). Interestingly, there was a small but significant difference in the *P. aeruginosa* biofilm density between PTFE tubing sections (*p* < 0.05), although the total variation equated to only ± 0.08 Log_10_ CFU cm^− 2^ and therefore has little impact on model use for investigating antimicrobial treatments.

When comparing the relative biofilm densities of the test species grown within the EBM, *P. aeruginosa* resulted in the highest biofilm density (7.63 ± 0.08 log_10_ CFU cm^− 2^), followed by *S. aureus* (6.96 ± 0.20 log_10_ CFU cm^− 2^), *E. coli* (5.49 ± 0.24 log_10_ CFU cm^− 2^) and *K. pneumonia* (5.33 ± 0.32 log_10_ CFU cm^− 2^). There is no significant difference between the density of *P. aeruginosa* biofilms and *S. aureus* biofilms cultured in the EBM, whereby these biofilm densities are significantly higher (*p* < 0.001) than that of the *K. pneumoniae* and *E. coli* biofilms (between which there was no significant difference in density). It is important to note that the same relative test section locations (i.e. test pieces) were used for both the control and NTP treatment groups during experimentation.

### Antimicrobial efficacy testing of the non-thermal plasma treatment system

**Fig. 4:**
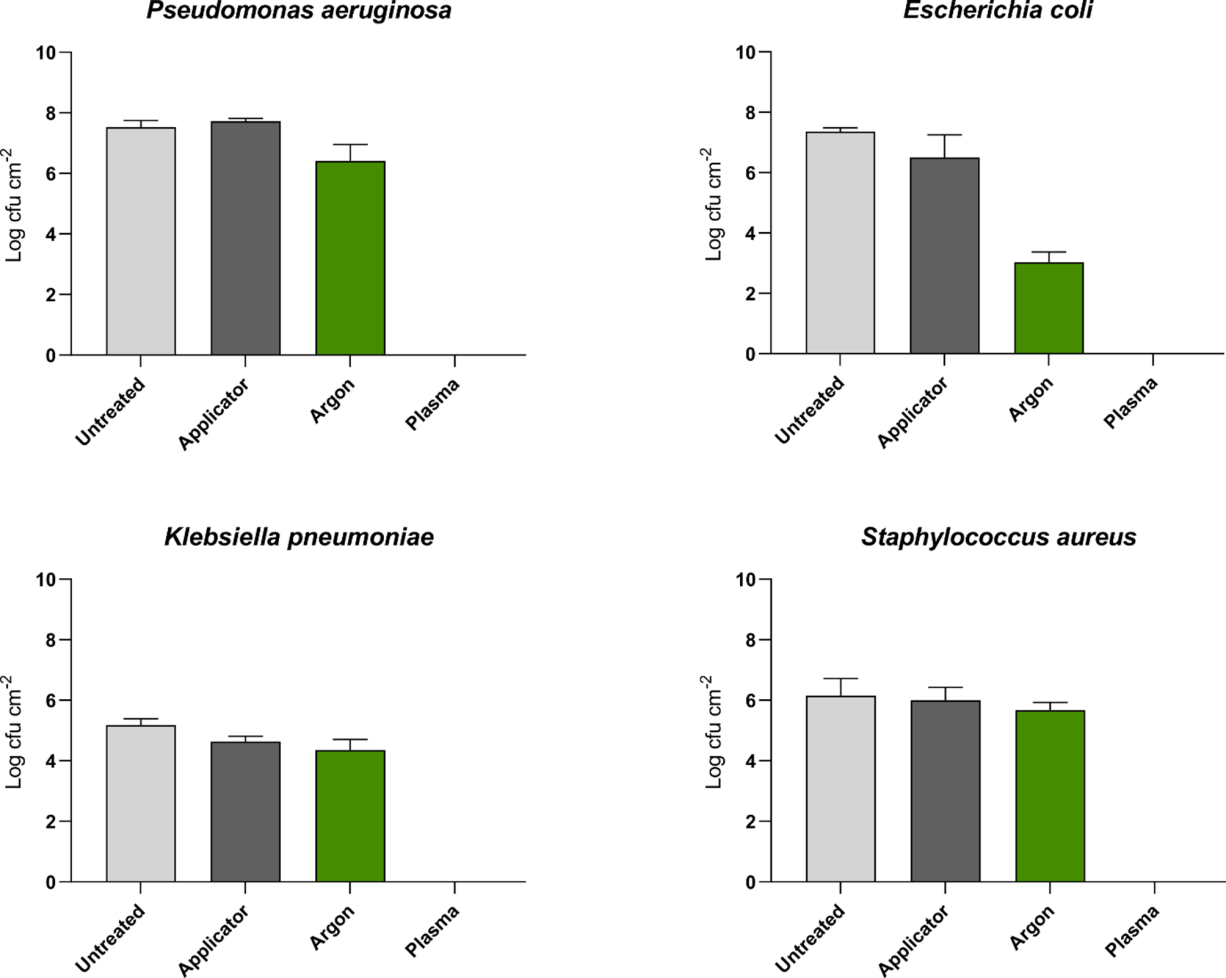
Antimicrobial efficacy of the NTP treatment system. *Pseudomonas aeruginosa*, *Escherichia coli*, *Klebsiella pneumoniae*, and *Staphylococcus aureus* biofilms grown for 72 h in the EBM and then subjected to control processes or non-thermal plasma treatment (*n* = 3 ± SD). Untreated = no treatment, tubing immediately sectioned for recovery. Applicator = control treated by advancing the applicator through the tubing at 100 mm min^- 1^ using the motorised advancer. Argon = control treated by advancing the applicator through the tubing at 100 mm min^- 1^ using the motorised advancer with the argon gas supply switched ON at a flow rate of 2.5 L min^- 1^. The argon control treatment resulted in a small but significant reduction in biofilm density likely as result of either direct gas pressure or the impact of desiccation of the top layer of bacteria as the gas dries out the surface. Plasma = non-thermal plasma treated by advancing the applicator through the tubing at 100 mm min-1 using the motorised advancer with the argon gas supply switched ON at a flow rate of 2.5 L min^- 1^ and ionised using a power setting of 80 W and duty cycle of 28%.

The antimicrobial efficacy of the NTP treatment system coupled with a prototype endoscope applicator (Fig. 2) was tested against biofilms cultured in the EBM for 72 h, whereby the results are shown in Fig. 4. The system was initially operated using a generator power setting of 80 W and a duty cycle of 28% (40 ms on, 100 ms off), with an argon gas flow rate of 2.5 L min^− 1^. The ‘applicator control’ (advancing the applicator without argon gas flow) had no significant effect on the recovered biofilm density of *P. aeruginosa*, *E. coli* or *S. aureus*, but resulted in a small but significant reduction in *K. pneumoniae* biofilm density (*p* < 0.05; 0.54 ± 0.152 Log_10_ CFU cm^− 2^). The ‘argon control’ resulted in a significant reduction (*p* < 0.01) in *P. aeruginosa*, *E. coli* and *K. pneumoniae* biofilms, but had no significant effect on *S. aureus* biofilm. NTP treatment of all four species of bacterial biofilms cultured within the EBM resulted in a significant reduction of recoverable viable bacterial cells to below the limit of detection for enumeration (< 0.9 Log10 CFU cm^− 2^). To determine if any viable cells had survived non-thermal plasma treatment, the experiment was repeated, and the replicate 1 cm PTFE tubing pieces transferred to universal bottles containing NB to assess whether there were any bacterial survivors, therefore assessing the high level disinfection capability of NTP treatment of PTFE tubing cultured within the EBM. Following 7 days of incubation, there was no bacterial growth in any universal bottles containing the NTP treated sections of PTFE tubing, demonstrating that the NTP treatment eliminated all viable biofilm cells. The bottles containing untreated controls, or treated with argon alone, all showed high levels of turbidity demonstrating bacterial growth. Consequently, overall mean log reductions for NTP treatment operated using a generator power setting of 80 W and a duty cycle of 28% (40 ms ON, 100 ms OFF), with an argon gas flow rate of 2.5 L min^− 1^ were 7.53 ± 0.12 Log_10_ CFU cm^− 2^ for *P. aeruginosa*, 7.36 ± 0.07 Log_10_ CFU cm^− 2^ for *E. coli*, 5.18 ± 0.12 Log_10_ CFU cm^− 2^ for *K. pneumoniae* and 6.16 ± 0.33 Log_10_ CFU cm^− 2^ for *S. aureus*.

### Optimisation of treatment parameters

The NTP system was optimised, to determine the most economical treatment parameters, i.e. the lowest power and gas flow rate that could be used while maintaining antimicrobial efficacy. The NTP generator power settings and then the argon gas flow rate was sequentially reduced and the antimicrobial efficacy tested against *P. aeruginosa* biofilms cultured for 72 h in the EBM. Table 1 shows the results of NTP treatment of *P. aeruginosa* biofilms using various power settings and argon gas flow rates. The lowest generator power setting and argon flow rate capable of producing a reliable plasma output was a power of 40 W with an argon gas flow rate of 2.0 L min^− 1^ (28% duty cycle), which still resulted in reduction in the recovered *P. aeruginosa* biofilm density to below the limit of detection for enumeration (Table 1) and removal of all viable cells was demonstrated through complete biofilm elimination testing. Testing these treatment parameters (40 W power, argon gas flow rate of 2.0 L min^− 1^) against biofilms of *E. coli*, *K. pneumoniae* and *S. aureus* cultured for 72 h in the EBM, confirmed the antimicrobial efficacy and complete removal of viable biofilm cells (through complete biofilm elimination testing). The following significant (*p* < 0.001) mean reductions in viable biofilm were observed; of 7.49 ± 0.05 Log_10_ CFU cm^− 2^ for *P. aeruginosa*, 5.07 ± 0.03 Log_10_ CFU cm^− 2^ for *E. coli*, 4.98 ± 0.27 Log_10_ CFU cm^− 2^ for *K. pneumoniae* and 7.13 ± 0.16 Log_10_ CFU cm^− 2^ for *S. aureus*.

**Table 1:** Optimisation of NTP treatment parameters against *P. aeruginosa* biofilms cultured in the EBM for 72 h showing Log_10_ reduction (cfu cm^-2^) of biofilm density compared to untreated controls and the result of complete biofilm elimination testing (via broth validation). ^a^Argon control, ^b^Untreated control. *reduced to below the limit of detection.

Power (W)	Argon flow rate (L min^− 1^)	Reduction (Log10 CFU cm^− 2^)	Complete Biofilm Elimination Test Result (where tested)
80	2.5	7.53^*^	No growth
70	2.5	7.49^*^	
60	2.5	7.49^*^	
50	2.5	7.49^*^	
40	2.5	7.64^*^	
40	2	7.49^*^	No growth
0^a^	2.5	1.12	Growth
0^b^	0	0	Growth

## Discussion

The bactericidal properties of NTP treatment systems when applied in a variety of settings, including the agri-food and dairy industries^24–26^ as well as in a medical context^28–30^, is well known, but this is the first study that has demonstrated that a NTP system coupled with a prototype endoscope applicator is effective for the complete removal of bacterial biofilms from the internal surface of PTFE tubing as a surrogate for endoscope operating channels. The current applicator has been designed with a maximum diameter of 2.8 mm (see Fig. 2b), which would enable its use for treatment of a wide range of standard adult endoscope and gastroscope operating channels. However, the applicator system could be further reduced in size (using smaller diameter microwave cable) to enable treatment of both air and water channels, as well as bronchoscopes and paediatric endoscope channels as required.

Various experimental biofilm models have been developed to represent the use and reprocessing of endoscopic devices. The biofilm model used within this study was adapted from a previously published method^33^ whereby the total culture time was reduced from six days to 72 h and the original inoculated input media (i.e. containing planktonic cells) was omitted with fresh sterile broth being used every 24 h. Daily input of fresh sterile media ensured that the biofilm developed from growth of attached cells rather than purely accumulation of planktonic cells from the circulating inoculum. Other biofilm endoscope methods have been developed to include organic material (also referred to as test soil) in recirculating media, and rinsing/cleaning steps are included to represent reprocessing procedures. For example, the studies by Alfa et al.^34,35^ used the artificial test soil as the recirculating media, which has been shown to mimic the organic contaminants that endoscopes are exposed to during clinical use. Therefore, for future studies it would be interesting to introduce a complex organic matrix into the sterile media replaced every 24 h as part of the EBM to see whether there is an impact on biofilm density and composition, and consequently the challenge afforded by this test method. Initial studies demonstrated biofilm formation within the EBM using the Crystal Violet staining biofilm assay (modified from Merritt, Kadouri and O’Toole^36^; data not shown). Subsequently, the actual biofilm density within the EBM was quantified (Fig. 3), and despite the reduced culture time the density of *E. coli* biofilms recovered from the internal lumen of PTFE tubing (5.49 ± 0.24 Log_10_ CFU cm^− 2^) exceeded the previously reported density of 4.49 Log_10_ CFU cm^− 2^^33^. This may be due to the increased nutrient availability provided by the input of fresh nutrient media or due to variations in the biofilm forming abilities of the strains used. This highlights the importance of verifying the performance of different strains and species of bacteria even when cultured in an established model system. Within this study, characterisation of biofilm formation throughout the PTFE tubing revealed little variation between tubing sections for all four test species, demonstrating the suitability of the model system for culturing consistent biofilms for subsequent experimentation. However, it should be noted that owing to the relatively short culturing time (72 h) the biofilm present within the EBM may constitute a relatively immature biofilm structure.

The control processes investigated within this study demonstrated that the action of passing the NTP applicator through the tubing lumen (with no argon gas or activation energy) resulted in a small statistically significant (*p* < 0.05) reduction in biofilm density for *K. pneumoniae* (although non-significant reductions were observed for other species). The addition of non-ionised argon gas at a flow rate of 2.5 L min^− 1^ for the ‘argon control’ showed a further significant reduction (*p* < 0.05) in biofilm density compared to untreated controls for all four species. This suggests the biofilms are differentially susceptible to both the shearing forces of the applicator passing through the lumen, and the argon gas flow, either related to direct gas pressure or the impact of desiccation of the top layer of bacteria as the gas dries out the surface. In fact, due to the experimental design employed, it is possible to disentangle the effects of the applicator and argon gas independently.

This study has demonstrated that NTP treatment of *P. aeruginosa*, *S. aureus*, *E. coli* and *K. pneumoniae* biofilms cultured within the EBM resulted in complete elimination from the PTFE tubing sections under the conditions tested (Fig .4). This included using a reduced generator power setting of 40 W with a reduced argon gas flow rate of 2.0 L min^− 1^, equating to significant reductions in density (*p* < 0.001) of between 7.49 ± 0.05 Log_10_ cfu cm^− 2^ and 4.98 ± 0.27 Log_10_ cfu cm^− 2^. Reducing the generator power setting and argon gas flow rate aimed to establish the most economical setting for running the non-thermal plasma treatment system while maintaining antimicrobial efficacy. The power and gas flow settings were reduced sequentially, to enable the treatment parameters to be optimised for complete removal of bacterial biofilm with the minimum necessary input of resources. Collectively, the testing protocol used within this study demonstrates that complete eradication of viable biofilm can be achieved using the developed NTP system coupled with a prototype endoscope applicator. Materials compatibility is an important aspect of new/novel decontamination approaches, and consequently it is important to note that within the context of this study no visible damage to the PTFE material surfaces was observed when using optical microscopy, but further research would be required to ensure that all endoscope components are compatible with non-thermal plasma treatment. In addition, it would be interesting to ascertain how much of the biofilm matrix (non-viable) remains after NTP treatment, and consequently future studies will include observing both the control and treated channels using scanning electron microscopy to observe the maturity of the biofilms formed within the EBM and any residual organic debris after treatment. This is important as any residual organic debris could act as both a growth substrate and potential scaffold for microbial colonisation, as well as potentially inhibiting effective cleaning and downstream disinfection/sterilization processes.

The antibiofilm effects of NTP observed within this study are supported by the wider literature, for example, a study successfully demonstrated the ability of a 5-minute NTP treatment to penetrate the full thickness of *E. faecalis* biofilms cultured on glass coverslips^29^. A similar study using confocal microscopy, but combined with mathematical modelling, showed a decrease in the thickness and volume of *S. aureus* and *P. aeruginosa* biofilms after NTP plasma treatment, combined with an increase in biofilm surface roughness and porosity, as well as a loss of biofilm biomass^37^. However, these studies did not quantify the reduction in biofilm density through culture of the surviving bacterial biofilm. A NTP generated using a gas mixture of 0.5% oxygen and 99.5% helium against *P. aeruginosa* biofilms (cultured within the Calgary biofilm device) demonstrated increased efficacy with increasing NTP treatment time, with the maximum treatment time of 4 min resulting in a 4 Log_10_ reduction in the recovered cell density^38^. In agreement with our study, complete biofilm elimination using Argon NTP treatment has also been observed against *Aggregatibacter actinomycetemcomitans* grown on titanium discs (a commonly used material for prosthetic implants), equating to a 6 Log_10_ CFU disc^− 1^ reduction relative to the untreated control discs^32^. Similarly, helium NTP treatment resulted in a 3 Log_10_ cfu mL^− 1^ reduction in *P. aeruginosa* biofilms on titanium disc surfaces grown within a CDC biofilm reactor^30^, but it was only when coupled with a chlorhexidine treatment that complete sterilisation of the titanium disc surface was achieved^30^. Non-thermal plasma has also been shown to have antimicrobial activity against mixed species biofilms of *P. aeruginosa* and *S. aureus* grown within a CDC biofilm reactor, although complete elimination was not observed within this study^39^. The antimicrobial mechanism of action of NTP is known to be multifaceted and comprises of a range of reactive species (including reactive oxygen and nitrogen species), electrons and ultraviolet photons^17,18^, which is one of the benefits of its use as an antimicrobial, although these are known to vary with the operating parameters of the configured NTP system^40^. Therefore, this will impact the antibiofilm efficacy, and explains the variance in the observed antimicrobial efficacy within the literature, coupled with differences in the target organisms and biofilm culture methods used. However, collectively it can clearly be concluded that NTP treatments are effective for reduction of bacterial biofilms, and in the case of the present study can result in complete biofilm elimination.

A recent study has also investigated the use of NTP for efficient disinfection of the inner channel of endoscope devices, using a two-step disinfection process coupling plasma activated water to cold atmospheric plasma treatment^40^. This demonstrated significant reductions of all the target microorganisms tested, including within mixed species biofilm models, although complete biofilm elimination was not observed using this system. Consequently, future development of the NTP system coupled with a prototype endoscope applicator should incorporate testing against mixed species biofilms within full-length PTFE channels, as well as treatment of clinically contaminated endoscopes to ensure the antibiofilm efficacy demonstrated within this study is replicable under real-world conditions. Endoscope operating channels are typically 300 cm in length, which with an applicator advancing rate of 100 mm min^− 1^ (tested within this study) would require a 30 min forward and 30 min reverse treatment time, adding 1 h to the total endoscope reprocessing time. In contrast, EO sterilisation results in an increase of 16 h to endoscope reprocessing times due to the lengthy aeration cycle required following EO exposure^41^, although this processing time is akin to automated LCS and VHP processes which are also more time efficient than EO sterilisation. Use of NTP treatment as the final step of endoscope reprocessing could provide an additional level of decontamination assurance without the excessive additional reprocessing time associated with EO sterilisation that would require a costly increase in the facilities endoscope inventory. NTP treatment is also a safer alternative to EO sterilisation, that does not leave behind any chemical residues that could pose a threat to the health of reprocessing facility staff^42^. Therefore, NTP treatment provides a faster and safer alternative to EO sterilisation for improving endoscope decontamination assurance. In comparison to LCS, appropriately designed NTP applicator treatment processes would be able to directly access complex geometry operating channels, and due to the fact that the NTP dries the channel during treatment, this approach would also not suffer from the same potential issues of residual liquid being present, particularly during VHP treatment which can result in treatment failure^14^. The end goal of this research would be to develop an automated device that would enable batch processing of endoscopes, through the use of multiple applicators that could be connected to a single non-thermal plasma generator and automatically advanced (via calibrated motors) through a range of endoscope devices in parallel. This would reduce the required labour time and ensure the cost effectiveness of the proposed approach.

Overall, the results of this study have demonstrated that a NTP system coupled with a prototype endoscope applicator is capable of sterilisation of surrogate endoscope operating channels contaminated with bacterial biofilms within a laboratory model system, consequently if this approach was applied within real world endoscopy settings it could help eliminate the risk of cross contamination between patients.

## Data Availability

The datasets generated and analysed within this study are available from the corresponding author on reasonable request.
